# Two Rieske Fe/S Proteins and TAT System in *Mesorhizobium loti* MAFF303099: Differential Regulation and Roles on Nodulation

**DOI:** 10.3389/fpls.2018.01686

**Published:** 2018-11-20

**Authors:** Laura A. Basile, Andrés Zalguizuri, Gabriel Briones, Viviana C. Lepek

**Affiliations:** Instituto de Investigaciones Biotecnológicas “Dr. Rodolfo A. Ugalde,” Universidad Nacional de San Martín, CONICET, Buenos Aires, Argentina

**Keywords:** Tat system, rhizobia, *Mesorhizobium*, Rieske protein, cytochrome bc_1_, nodulation

## Abstract

*Mesorhizobium loti* MAFF303099 is a rhizobial strain that nodulates *Lotus* spp. A *M. loti* MAFF303099 mutant strain affected in the *tatC* gene was generated. This strain presented an altered protein secretion level to the culture supernatant and also a higher sensitivity to SDS. Its nodulation phenotype on *Lotus* showed the induction of small and colorless nodules, and in a larger number than those induced by the wild-type strain. In addition, these nodules presented defects in the degree of occupation by rhizobia. Two Rieske Fe/S proteins, encoded by the *mll2707* and *mlr0970* genes, were predicted as potential Tat substrates in *M. loti* MAFF303099. The transcriptional expression of *mll2707* and *mlr0970* genes was analyzed under different oxygen growth conditions. The *mll2707* gene was expressed constitutively, while the expression of the *mlr0970* gene was only detected under anaerobic and microaerophilic *in vitro* conditions. Both genes were down-regulated in the *tatC* mutant strain. *mll2707* and *mlr0970* mRNAs from the wild-type strain were detected in nodules. Using a translational reporter peptide fusion, we found that the Mll2707 protein was only detectable in the wild-type strain. On the other hand, although Mlr0970 protein was detected in wild-type and *tatC* mutant strains, its association with the membrane was favored in the wild-type strain. The *tatC* and the *mll2707* mutant strains were affected in the cytochrome c oxidase activity. These results confirm that Mll2707 is required for cytochrome c-dependent respiration and that Tat functionality is required for the correct activity of Mll2707. The *mll2707* mutant strain showed a nodulation phenotype similar to the *tatC* mutant strain, although it presented only a slight difference in comparison with wild-type strain in terms of nodule occupation. No defective phenotype was observed in the nodulation with the *mlr0970* mutant strain. These results indicate that, of the two Rieske Fe/S proteins coded by *M. loti* MAFF303099, only Mll2707 expression is required for the induction of effective nodules, and that the functionality of the Tat system is necessary not only for the correct function of this protein, but also for some other protein required in an earlier stage of the nodulation process.

## Introduction

The symbiotic process given by the interaction between rhizobia and legumes leads to the biological fixation of atmospheric nitrogen, which is performed inside structures formed at plant roots called nodules. The nodulation process is the result of a sequence of steps that begins with bacterial attachment to the plant root, and that continues with the induction of mature active nodules. Inside these nodules bacteria differentiate into bacteroides, adapting their respiratory electron transport chain in order to cope with the micro-oxic environment within the nodule, a condition strictly required for normal nitrogenase activity. It was determined that Rieske/cytochrome bc_1_ complex is a key component of the rhizobial respiratory chain and that is essential for symbiotic nitrogen fixation (Thöny-Meyer et al., 1989; Wu et al., 1996; Pickering et al., 2012). Rieske/cytochrome bc_1_-type complex is encoded in a gene cluster that contains an ubiquinol cytochrome c reductase iron-sulfur subunit (or Rieske Fe/S protein), a cytochrome b-type protein, and a cytochrome c1-type protein, although in some species the cluster does not code for a cytochrome c-type protein (Ten Brink et al., 2013). The Rieske/cytochrome bc_1_ complex performs the electron transfer from membrane-diffusing quinols to small soluble or membrane-bound redox proteins and thereby participates in the generation of a proton motive potential (Duval et al., 2010). It was described that the mutation of the Rieske Fe/S protein affects the nitrogen fixing activity in symbiosis in the case of *Bradyrhizobium japonicum* (Thöny-Meyer et al., 1989) and *Sinorhizobium meliloti* (Pickering et al., 2012). To date, all the rhizobia in which the Rieske/cytochrome bc_1_ complex and its involvement in the nitrogen fixation process were studied, present only one cluster of genes coding for this complex. However, in several bacteria more than one Rieske Fe/S protein (Schneider and Schmidt, 2005) or more than one cluster of genes coding for Rieske/cytochrome bc_1_ complex (Ten Brink et al., 2013) have been found. Presence of multiple copies could be the result of gene duplication or a lateral gene transfer event (Ten Brink et al., 2013). It has been suggested that the existence of multiple copies of Rieske Fe/S proteins allow an organism to adapt their electron transfer chains to changing environmental conditions (Schneider and Schmidt, 2005).

The Rieske Fe/S protein was described to be transported to the inner membrane through the twin-arginine translocation (Tat) system (Bachmann et al., 2006). This translocation system mediates the transport of folded proteins across the inner membrane to the periplasm space. Tat machinery is composed of three membrane proteins that in bacteria are termed TatA, TatB, and TatC (Frobel et al., 2012a). Enterobacteria contain an additional paralog of TatA, called TatE that can functionally replace TatA (Frobel et al., 2012a). The TatA and B proteins are predicted to contain one transmembrane α-helix, while the TatC protein has six predicted transmembrane helices and has been proposed to function both as the principal component of the translocation channel and as a receptor for preproteins (DeLisa et al., 2002). Mutagenesis of either TatB or C completely abolishes export (Sargent et al., 1998; Lavander et al., 2006). Proteins translocated by the Tat system harbor a twin arginine signal motif Z-R-R-X-φ-φ in the N-terminal sequence, where Z is a polar residue, X is any residue and φ are hydrophobic residues (Frobel et al., 2012b; Simone et al., 2013). Most Tat substrates are soluble proteins released after membrane passage, however, some remain anchored by either N- or C-terminal transmembrane domains (Frobel et al., 2012a). In the case of Rieske Fe/S protein the N-terminus serves a dual role as export signal and membrane anchor (Bachmann et al., 2006) while the C-terminal hydrophilic domain is exposed in the periplasmic space (De Buck et al., 2007). Tat disruption in *S. meliloti* affects cell viability (Pickering and Oresnik, 2010). The absence of a functional Tat system in *Rhizobium leguminosarum* bv. viciae UPM791 strain induces the formation of white nodules unable to fix nitrogen (Meloni et al., 2003). A defect to induce active nodules was also described for a *R. leguminosarum* bv. viciae 3481 *tat* mutant strain (Krehenbrink and Downie, 2008).

*Mesorhizobium loti* specifically nodulates *Lotus* spp. *M. loti* MAFF303099 (recently reclassified as *M. japonicum* MAFF303099) (Martínez-Hidalgo et al., 2016) genome was completely sequenced (Kaneko et al., 2000) being identified several genes coding for different protein secretion systems (Schmeisser et al., 2009). To date, only *M. loti* MAFF303099 type three secretion system (T3SS) has been characterized (Sánchez et al., 2009, 2012; Okazaki et al., 2010). In the present work we characterized the Tat system in *M. loti* MAFF303099. In addition we described the existence of two clusters of genes coding for the Rieske/cytochrome bc_1_-type complex, and analyzed its relation with the Tat system and its involvement in the nodulation process on *Lotus tenuis*.

## Materials and Methods

### Plasmids, Bacterial Strains, and Growth Media

Bacterial strains and plasmids used in this study are listed in Supplementary Table S1. *Escherichia coli* strains were grown at 37°C in Luria-Bertani media. *M. loti* MAFF303099 strains were grown at 28°C in AB minimal medium (Douglas et al., 1985) supplemented with sucrose (0.5% w/v) or rich TY medium. When necessary, antibiotics were added to the following final concentrations: gentamicin (Gm), 30 μg/ml; ampicillin (Amp), 100 μg/ml; kanamycin (Km), 50 μg/ml for *E. coli* or 200 μg/ml for *M. loti* MAFF303099; and tetracycline (Tc), 10 μg/ml for *E. coli* or 1 μg/ml for *M. loti* MAFF303099. For aerobic *in vitro* incubation, cultures were grown in plates for 3 days; microaerophilic conditions were simulated by plating on AB 1.6% agar plates and covering with 12 ml of AB 0.3% agar; anaerobic *in vitro* incubation was performed in an anaerobic jar using an AnaeroGen sachet (OXOID). Microaerophilic and anaerobic plates were incubated for 5–7 days.

### Construction of *M. loti* MAFF303099 Mutant Strains

Oligonucleotides and plasmids used for mutant construction are listed in Supplementary Table S1. For non-polar *tatC* mutant construction, a Gm cassette without transcriptional termination sequence (Ugalde et al., 2000) was introduced interrupting the coding region of the gene. Briefly, *tatC* gene was amplified by PCR and cloned into T-Easy vector. A Gm cassette was introduced by cutting with the appropriate restriction endonuclease enzymes. The resulting construction (*tatC*::Gm) was subcloned into pK18mobTc plasmid (Sánchez et al., 2009) and used to transform *E. coli* S17 competent cells. The plasmid was then introduced by biparental conjugation into *M. loti* MAFF303099. Double recombinant cells were selected by testing sensitivity to Gm and Tc. The double crossover event was confirmed by PCR. The same strategy, with the Gm cassette without transcriptional termination sequence and the pk18mobTc, was used for non-polar *mll2707* mutant strain generation. For *mlr0970* mutation, a Tc cassette and the pK18mob vector (Schäfer et al., 1994) were used instead. The construction was made by amplifying a region upstream and a region downstream *mlr0970* gene. Plasmid pGEM-T-easy containing both fragments in the correct adjacent orientation was selected, and a Tc cassette was introduced between them. The construction (*mlr0970*::Tc) was subcloned into pK18mob vector, used to transform competent *E. coli* S17 cells, and introduced by biparental conjugation into *M. loti* MAFF303099. Double recombination event was selected by testing sensitivity to Tc and Km. For the double mutant, the *mll2707* mutant strain was used as the receptor strain in the biparental conjugation with *E. coli* S17 cells containing the 0970::Tc construction cloned into pK18mob vector. For the complementation of the *tatC* mutant strain, the entire sequence of TatC with its RBS was cloned into the pBBR1MCS-2 vector under *lacZ* promoter region, and introduced by conjugation into the *tatC* mutant strain. Recombinant cells were selected by Km resistance and the presence of the entire *tatC* gene was checked by PCR.

### SDS Sensitivity Assays

Two milliliters of TY medium containing a range of concentrations of SDS were inoculated with *tatC*, complemented *tatC*, or wild-type *M. loti* MAFF303099 strains at OD_600_ of 0.05. Cultures were incubated at 28°C with agitation (250 rpm) for 22 h and OD_600_ was measured. Around 100% survival was defined as OD of each strain grown in absence of SDS. At least two independent experiments were performed in duplicate cultures.

### Isolation of Extracellular and Periplasmic Proteins

Extracellular proteins were obtained from 100 ml supernatants of late exponential phase culture. Bacterial free medium was obtained by centrifugation as described in Sánchez et al. (2009). Supernatant proteins were precipitated with 10% trichloroacetic acid (TCA) and washed with acetone 80%-Tris–HCl 50 mM pH 8. Periplasmic proteins were obtained from 1.5 ml of late exponential phase cultures by the chloroform method as described in Ames et al. (1984). Samples were resuspended in cracking buffer plus 2% β-mercaptoethanol or DTT (100 mM) and boiled for 5 min. Proteins were separated by SDS–PAGE in a 12 or 15% polyacrylamide gel and stained using silver nitrate.

### Analysis of Transcriptional Expression of *mll2707* and *mlr0970* by Real Time RT-PCR Assays

Total RNA was extracted from *M. loti* MAFF303099 cultures grown in solid AB medium under oxygenated, microaerophilic, or anaerobic conditions, or from nodules developed on *L. tenuis* plants. Bacteria were recovered from plates after 5 days of incubation (or 3 days in the case of aerobic cultures) and resuspended in TE buffer; RNA was extracted from 1 ml suspension of OD_600_ 2.5. Samples were centrifuged and resuspended in 1 ml TRIzol reagent (Invitrogen – Life Technologies). Forty nodules from *L. tenuis* plants (inoculated with *M. loti* MAFF303099 wild-type strain) were collected at 45 dpi, frozen immediately in liquid nitrogen, and stored at -70°C. For RNA extraction, nodules were grinded until powder in a mortar adding liquid nitrogen to keep samples frozen. One milliliter of TRIzol reagent was added. RNA purification was performed by the TRIzol method according to the manufacturer’s instructions.

RNA quality was evaluated by agarose gel electrophoresis in presence of 1 vol. formamide. RNA samples (about 600 ng of total RNA) were subjected to DNAse treatment (RQ1 RNase-Free DNase – Promega) and reverse transcription with random primers (SuperScript II Reverse Transcriptase – Invitrogen) according to the manufacturer’s instructions. Primers used in RT-PCR real time assays are listed in Supplementary Table S1. Real time PCR assays were performed with SYBR-GREEN Fast-Master Mix (Bioline) in a 7500 Detection System (Applied Biosystems). RNA samples isolated from at least two independent experiments were tested in triplicate for each condition. The relative amounts of each target mRNA were calculated as described in Pfaffl (2001) using *sigA* and *rpoA* as reference genes (Alexandre et al., 2014). For the calculation of *mlr6630* and *mll6411* expression levels the Delta-Delta C_T_ method (Livak and Schmittgen, 2001) was used, taking the aerobic growth as the reference condition. Standard deviations were calculated by error propagation.

### Construction of 3X FLAG Translational Fusions

The complete sequences coding for *mll2707* and *mlr0970* (without the STOP codon) were amplified by PCR and cloned separately into pBAD 3X FLAG vector. The fragment containing the fusion to the 3X FLAG sequence at the C-term was cut with the appropriate restriction enzymes and subcloned into pBBR1MCS-4 vector under the regulation of *lac* promoter. The resulting plasmids were transferred to *M. loti* MAFF303099 wild-type and *tatC* mutant strains by triparental mating. The construction *mll2707* fused to 3XFLAG in pBBR1MCS-4 was also used to complement the *mll2707* mutant strain (strain *mll2707* plus Mll2707). For the complementation of the *tatC* mutant stains containing the reporter fused proteins, the entire sequence of TatC cloned in the pBBR1MCS-2 vector was introduced. The resulting strains (*tatC* 0970-flag-plus TatC, and *tatC* 2707-flag-plus TatC) were grown in presence of both antibiotics (Amp, Km), for the maintenance of both plasmids. Oligonucleotides and strains used are listed in Supplementary Table S1. Restriction endonuclease sites were incorporated into forward and reverse primers for cloning strategies.

### Isolation of Membrane and Cytoplasmic Proteins and Western Blot Analysis

Bacterial membranes were isolated from 50 ml of late exponential phase cultures by cellular lysis using osmotic shock as described in Mercante et al. (2015). After ultracentrifugation of 4 h at 40,000 × *g* (70 Ti rotor, Beckman), the pellet containing membrane proteins was resuspended in cracking buffer. The supernatant containing cytoplasmic proteins was precipitated with TCA as previously described. Proteins were separated by SDS–PAGE. Immunoblotting was performed using mouse anti-FLAG M2 monoclonal antibody (Sigma) in the Li-Cor Odyssey equipment. As a control of protein charge rabbit anti-Omp19 polyclonal antibody was used (Mercante et al., 2015). Anti-mouse and anti-rabbit fluorescent antibodies were used for detection in the Li-Cor Odyssey equipment. Analysis of relative band intensity was made using the Odyssey software.

### Analysis of Cytochrome c Oxidase Activity

Cytochrome c oxidase activity was assayed using oxidase disks (Britannia) impregnated with *N*,*N*-dimethyl-*p*-phenylenediamine (oxalate). The disks were immersed in 60 μl of bacterial culture according to the manufacturer’s instructions. Color development was analyzed within the first 1 min.

### Nodulation Assays

*Lotus tenuis* seeds were surface-sterilized and pregerminated. Nodulation was observed by the agar slant method (Vincent, 1970). Three-day-old seedlings were placed into column tubes containing agar B&D 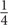 (Broughton and Dilworth, 1971), inoculated with *M. loti* MAFF303099 wild-type and mutant strains at OD_600_ of 0.8 (10 μl/root), and observed daily for nodule number. Results correspond to the average of three experiments of 10 plants each. Dry weight was determined by drying plant stems at 60°C for 24 h. Statistical analysis was carried out by one way ANOVA or Student’s *t*-tests.

### Determination of Nodule Occupancy

For each *M. loti* MAFF303099 strain analyzed, six nodules were surface sterilized by subsequent 1 min incubations in 2% sodium hypochlorite and 15% hydrogen peroxide, and then rinsed three times with sterile distilled water, as described in Sturz et al. (1997). Nodules were then crushed, diluted, and individually plated on AB plates. Plates were incubated at 28°C and the number of colonies developed was counted. Statistical analysis was performed by Kruskal–Wallis and Mann–Whitney pairwise comparisons.

## Results

### Phenotypic Characterization of *M. loti tatC* Mutant Strain

**FIGURE 1:**
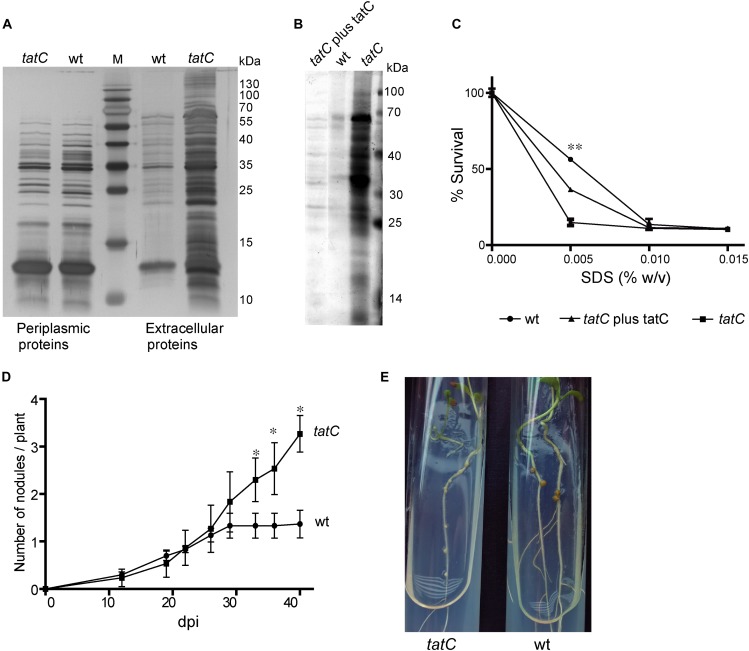
Phenotypic characterization of *tatC* mutant strain. **(A)** Periplasmic and extracellular proteins were separated in a 12% SDS–PAGE and stained with silver nitrate. **(B)** SDS–PAGE of extracellular proteins from wild-type (wt), *tatC*, and complemented *tatC* (*tatC* plus tatC) mutant strains. **(C)** SDS sensitivity assay. Bacterial survival was determined in a range of concentrations of SDS, were 100% survival is defined as OD_600_ of each strain grown in absence of SDS. Error bars correspond to standard deviations from replicates in duplicate cultures. Asterisks correspond to a Student’s *t*-test between wild-type and *tatC* mutant strains at 0.005% w/v of SDS and indicate a *p*-value <0.01 (^∗∗^). **(D,E)** Nodulation assays in *Lotus tenuis* plants inoculated with *M. loti* wild-type and *tatC* mutant strains. **(D)** Number of nodules by plant, dpi: days post inoculation. Experiments were made in triplicate of 10 plants each. Errors bars correspond to standard deviations from replicates. Asterisks indicate a *p*-value <0.05 (^∗^) in Student’s *t*-tests. **(E)** Photography of nodules developed.

The *M. loti* MAFF303099 *mll1082* gene codes for a predicted TatC protein. Downstream, is the *msl1085* gene that codes for a TatA/E-type protein. Between them, we identified an open reading frame coding for a 252 aa polypeptide (Supplementary Figure S1) that shares a 86–93% of identity with TatB from *M. loti* TONO, *M. loti* NZ82037 and *M. huakuii* 7653R. Given the central role of TatC in the normal function of the Tat secretion system, a *tatC* mutant was generated in order to analyze the effect of a mutation in the Tat system of *M. loti* MAFF303099. The *tatC* mutant strain presented a culture growth curve similar to the wild-type strain (not shown). A protein electrophoretic assay was performed to evaluate potential modifications in the secreted protein profile, both in the periplasmic space and in the culture supernatant. The *tatC* mutant strain showed higher amount of secreted proteins to the supernatant compared to the wild-type, with no differences in the protein periplasmic profile neither qualitative nor quantitative (Figure 1A). Complementation of *tatC* mutant strain with TatC protein, restores the supernatant protein levels similar to the wild-type phenotype (Figure 1B). An analysis of sensitivity to SDS was carried out in order to analyze a possible defect on membrane integrity that could explain the massive release of proteins to the supernatant observed in the mutant strain. A lower survival percentage in cultures grown in presence of low SDS concentrations was observed in the case of the mutant strain a defect that was largely restored in the complemented strain (Figure 1C). These data suggest that *tatC* mutation altered membrane integrity. Next, a nodulation experiment was performed in order to study if *tatC* mutant also displays defects in the interaction with its host *L. tenuis*. As shown in Figure 1D, *tatC* mutant strain induced a greater number of nodules than the wild-type strain, although they were colorless and smaller than those induced by the wild-type strain (Figure 1E). The induction of increased number of white and small nodules is indicative of defects in the nitrogen fixation process (D’Antuono et al., 2005). This defect was consequently reflected on the dry weight of the plants. A significant lower value was determined in plants inoculated with the *tatC* mutant strain (Table 1) compared to those inoculated with the wild-type one. Wild-type nodulation phenotype was restituted in the complemented strain, which elicited normal nodules (data not shown).

**Table 1:** Dry weight of plants expressed as percentage relative to plants inoculated with the wild-type strain (100%).

Strain	Dry weight % (relative to wild-type strain)	
*tatC*	77.59 ± 6.39	^∗∗^
*2707/0970*	86.82 ± 7.67	ns
*2707*	87.33 ± 4.24	ns
*0970*	103.11 ± 7.46	ns
Non-inoculated	66.43 ± 6.08	^∗∗∗^


*All experiments were performed in triplicate of 10 plants each. Asterisks indicate significant differences in dry weight to plants inoculated with the wild-type strain in Student’s *t*-tests. ^∗∗^Indicate significant differences at *p* < 0.01, ^∗∗∗^*p* < 0.001, ns, no significant differences.*

### *In silico* Identification of Potential Substrate Proteins of the Tat System

*Mesorhizobium loti* MAFF303099 proteins carrying the twin arginine signal motif in the N-terminus were identified by the Pred-TAT method (Bagos et al., 2010). Two ubiquinol-cytochrome C reductase iron-sulfur subunits (Rieske Fe/S proteins) were identified as putative Tat substrates, encoded by *mll2707* and *mlr0970* genes (Supplementary Table S2). Whereas other rhizobia have been described to present a single copy of this gene (Thöny-Meyer et al., 1989; Wu et al., 1996; Pickering et al., 2012), *M. loti* MAFF303099 presents two different Rieske Fe/S proteins encoded by two different Rieske/Cyt bc_1_-type complex gene clusters. As shown in Supplementary Figure S2A, there is an ORF coding for a cytochrome b protein placed downstream the *mlr0970* gene and a tandem of two genes coding for cytochrome b and cytochrome c_1_ placed downstream the *mll2707* gene. Both Rieske Fe/S proteins share a sequence identity of 79% (Supplementary Figure S2B) and are predicted to be High Potential iron/sulfur proteins as they have a serine and a tyrosine residues in conserved positions (Baymann et al., 2012).

### Analysis of the Transcriptional Expression of the Two *M. loti* Rieske Fe/S Proteins

**FIGURE 2:**
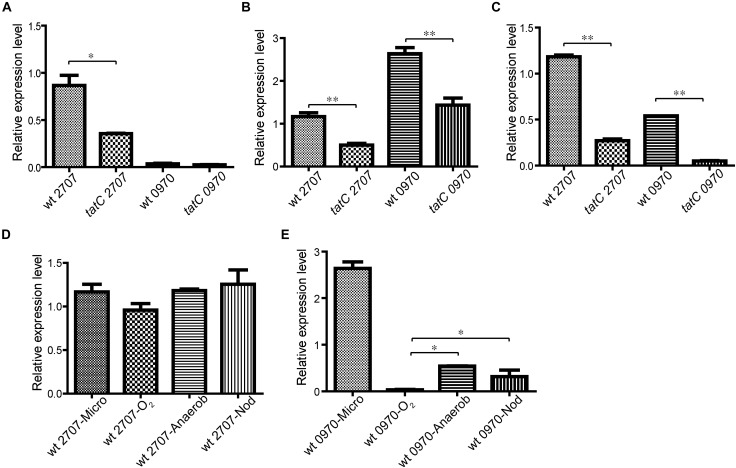
Analysis of *mll2707* and *mlr0970* gene expression in *M. loti* MAFF303099 wild-type and *tatC* mutant strains. The expression level of *mll2707* and *mlr0970* genes was determined by real time RT-PCR assays. Below each bar the strain and the gene assayed are indicated. Data correspond to triplicates in at least three independent experiments. mRNA was extracted from wild-type (wt) and *tatC* mutant strains grown in solid media under aerobiosis (O_2_) **(A)**, microaerophilic conditions (Micro) **(B)**, and anaerobiosis (Anaerob) **(C)**. **D,E** Comparative relative expression level of *mll2707* **(D)**, and *mlr0970* **(E)** genes in the wild-type strain growing under the different oxygen conditions *in vitro* and in nodules (Nod). Errors bars correspond to standard deviations from replicates. Asterisks indicate a *p*-value <0.05 (^∗^) or 0.01 (^∗∗^) in Student’s *t*-tests.

The transcriptional expression level of *mll2707* and *mlr0970* genes was studied by real time RT-PCR. Expression analysis was made in aerobic, microaerophilic, and anaerobic conditions in both wild-type and *tatC* mutant strains. All the assays were made on bacteria growing on solid media. To reduce the oxygen available to the bacterial culture, a system of two agar layers was used for microaerophilic growth as described in Section “Materials and Methods.” For controlled anaerobic conditions an anaerobic jar was used. Figure 2A shows that under aerobic conditions only *mll2707* gene expression was detected, being higher in the wild-type strain than in the *tatC* mutant strain. The same expression pattern was observed in microaerophilic and anaerobic conditions for *mll2707* gene (Figures 2B,C). *mlr0970* gene expression was induced only under microaerophilic and anaerobic conditions and, as occurs with *mll2707*, its expression was also affected by the *tat* mutation (Figures 2B,C). Analysis of *mll2707* and *mlr0970* transcriptional expression in nodules was performed for the wild-type strain at 45 days post inoculation. Figures 2D,E show the relative expression of both genes at the different oxygen growing conditions. The *mll2707* expression was constitutive at all the different conditions assayed (Figure 2D). The *mlr0970* expression occurs only under restricted *in vitro* oxygen conditions being also detectable in nodules (Figure 2E).

The expression level of *mlr6630* and *mlr6411* genes was assayed as positive controls of microaerophilic and anaerobic conditions (Supplementary Table S3). These genes code for two cytochrome oxidase c proteins, present outside and inside the *M. loti* MAFF303099 symbiotic island, respectively, and have previously been reported to be induced under low oxygen concentration (Uchiumi et al., 2004).

A more detailed analysis of the *mlr0970* promoter region showed a sequence similarity to the motif TTG-N_8_-CAA recognized by the Fnr-type transcriptional regulators which control the expression of genes for micro-oxic respiration (Bueno et al., 2012; Supplementary Figure S3).

### Analysis of Rieske Fe/S Proteins

**FIGURE 3:**
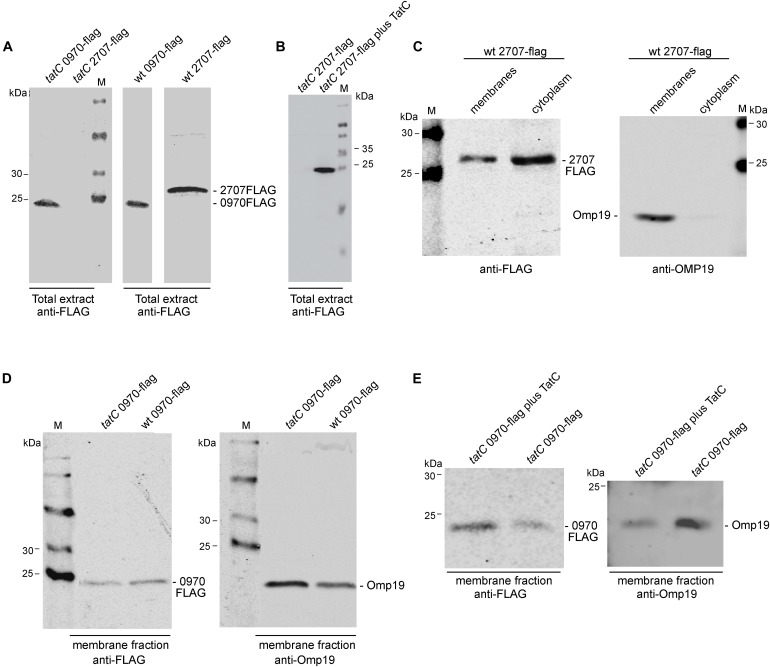
Detection of constitutively expressed Mll2707 and Mlr0970 fusion proteins. Western blot of *M. loti* MAFF303099 strains containing the *mll2707* or *mlr0970* genes fused to a 3XFLAG in an expression vector (pBBRMCS-4, Amp^r^). Proteins were separated by 12% SDS–PAGE (except for Figure 3B, in which a 15% polyacrylamide gel was used) and then immuno-bloted and probed with anti-FLAG and anti-Omp19 antibodies. **(A,B)** Total bacterial extracts. **(C–E)** Subcellular fractions obtained after an osmotic shock and ultracentrifugation protocol. M, protein marker. Positions of size markers loaded onto the gel are labeled (in kDa). For each strain specification see Supplementary Table S1.

**FIGURE 4:**
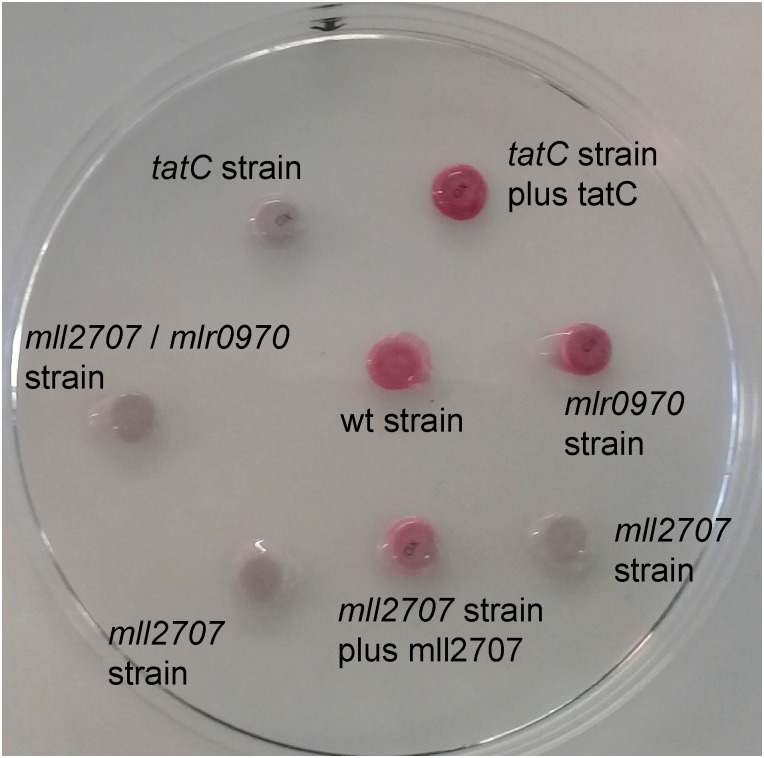
Cytochrome oxidase activity in *M. loti* MAFF303099 wild-type, mutant, and complemented mutant strains. Disks impregnated with *N*,*N*-dimethyl-*p*-phenylenediamine (oxalate) were added to 60 μl of wild-type (wt), *tatC*, *mll2707*, *mlr0970*, and *mll2707/mlr0970* mutant strains, *tatC* mutant strain complemented with *tatC* gene cloned in pBBR1MCS-2 (*tatC* strain plus tatC), and *mll2707* mutant strain complemented with *mll2707* gene in pBBR1MCS-4 (*mll2707* strain plus mll2707) (in this case the mll2707-3XFLAG construction was used for complementation). Development of a pink coloration was evaluated within 1 min.

In order to analyze the stability and cellular localization of both Rieske Fe/S proteins, translational fusions to the reporter epitope 3XFLAG were created. The 3XFLAG sequence was fused in frame to the C-terminal of each Rieske Fe/S gene. Constructs were cloned under the constitutive promoter *Plac* of the pBBR1MCS-4 plasmid and introduced by conjugation into wild-type and *tatC* strains. As shown in Figure 3A Mll2707 and Mlr0970 reporter fused proteins were expressed in the wild-type strain. Surprisingly, when these constructions were introduced into the *tatC* mutant strain, only Mlr0970 was detected by Western blot analysis (Figure 3A). Restoration of a copy of the *tatC* gene cloned in the pBBR1MCS-2 restituted the band corresponding to the Mll2707 reporter fused protein (Figure 3B), suggesting that a functional TAT system is required for the stability of Mll2707 protein or mRNA. The theoretical molecular weights of Mll2707 and Mlr0970 proteins fused to the reporter are 22.87 and 21.69 kDa, respectively; in a 12% polyacrylamide gel they were observed above and below the position corresponding to the marker of 25 kDa, respectively (Figure 3A). In the Figure 3B the relative position of Mll2707 fused protein respect to the 25 kDa marker was observed slightly different due to the different polyacrylamide gel percentage. To determine the cellular localization, membrane and cytoplasmic fractioning was performed. Both proteins were detected, besides the cytoplasmic fraction, associated to the membranes (Figure 3C and Supplementary Figure S4). In the case of the Mlr0970 reporter fused protein a higher level was observed in the cytoplasmic fraction comparing to the membrane fraction in the *tatC* mutant strain, in difference to the relative levels observed for the wild-type strain (Supplementary Figure S4). To confirm that the Mlr0970 protein was affected in its subcellular localization we compared wild-type and *tatC* strains in a simultaneous assay using anti-FLAG and anti-Omp19 antibodies to discard differences due to a loading effect (Mercante et al., 2015; Figure 3D). When comparing the intensity of Mlr0970 reporter band in membranes of wild-type and *tatC* mutant strains, relative to Omp19 loading marker, it was noted that it was higher in the wild-type strain (Figure 4D, approximately a threefold increase, determined by using the Li-Cor Odyssey software). In another independent experiment, this difference was about six times (data not shown). Restoration in the mutant strain of a copy of the *tatC* gene cloned in the pBBR1MCS-2, restituted the wild-type phenotype of Mlr0970 reporter protein localization relative to the mutant strain (Figure 3E, approximately a fivefold increase of Mlr0970 reporter fused protein levels in membranes of the complemented mutant strain respect to the membranes of the *tatC* mutant). These data suggest that the TatC protein is involved in the correct localization of the Mlr0970 protein.

### Analysis of Cytochrome bc_1_ Functionality

To analyze the functionality of cytochrome bc_1_ complex in *M. loti* MAFF303099 wild-type and mutant strains, a cytochrome c oxidase assay was performed on bacterial cultures as described in Section “Materials and Methods” (Meloni et al., 2003). Using *N*,*N*-dimethyl-*p*-phenylenediamine (oxalate) as the substrate, the development of a pink coloration is indicative of a positive result. Figure 4 shows that the wild-type and the *mlr0970* mutant strains were positive, whereas the *tatC* and *mll2707* mutant strains were negative for oxidase activity. These results indicate that, in vegetative cells of *M. loti* MAFF303099, the functionality of cytochrome bc_1_ depends on the functionality of Mll2707, which is in concordance with the absence of expression of *mlr0970* gene under aerobic conditions. On the other hand, the requirement of TatC for cytochrome oxidase activity is consistent with the above results showing that transcriptional expression of *mll2707* gene, and stability of Mll2707 reporter protein or mRNA were upon the presence of a functional Tat system. Cytochrome oxidase activity was restituted in the complemented *tatC* and *mll2707* mutant strains (Figure 4).

### Characterization of the Nodulation Phenotype of Rieske Fe/S Mutants

**FIGURE 5:**
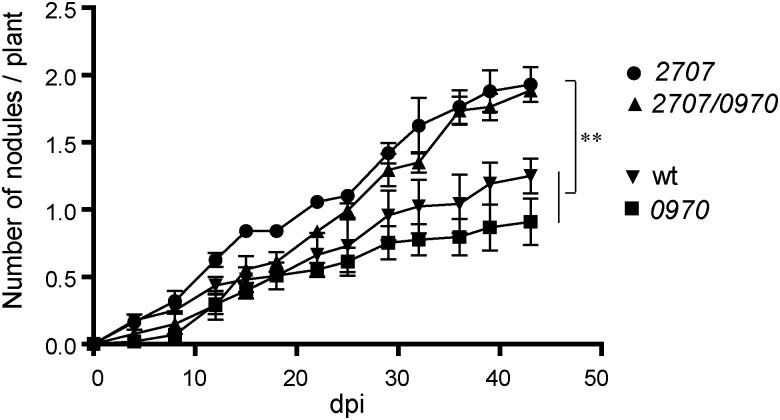
Nodulation phenotype of *M. loti* MAFF303099 Rieske mutants. *Lotus tenuis* plants were inoculated with wild-type (wt) and Rieske mutant strains: *mll2707*, *mlr0970*, or *mll2707*/*mlr0970* double mutant. dpi: days post inoculation. All experiments were performed in triplicate of 10 plants each. Error bars correspond to standard deviations from replicates. One way ANOVA was performed at 43 dpi, asterisks indicate a *p*-value <0.01 (^∗∗^) in Tukey contrasts.

In order to determine a potential role of *M. loti* MAFF303099 Rieske Fe/S proteins in the nodulation process, mutants affected in *mll2707* gene, *mlr0970* gene or both genes were generated. A nodulation kinetic analysis was performed on *L. tenuis* plants. Similar to *tatC* mutant phenotype, *mll2707* and *mll2707*/*mlr0970* mutant strains induced smaller and colorless nodules, and in a higher number compared to the wild-type strain (Figure 5). The single *mlr0970* mutant, in contrast, showed a similar nodulation kinetic to that observed for the wild-type strain (Figure 5). Nodules presented also the same color and size than those induced by the wild-type strain indicating an active nitrogen fixation activity (data not shown). The dry weight of the plants was determined at 45 days post inoculation. The plants inoculated with *mll0970* single mutant strain showed similar dry weight values that those inoculated with the wild-type strain confirming that Mlr0970 protein functionality is not relevant for the induction of effective symbiosis (Table 1). On the other hand, although not significant, plants inoculated with *mll2707* and *mll2707/mlr0970* mutant strains showed a lower value of organic matter than those inoculated with the wild-type strain (Table 1). Indeed, the difference obtained between plants inoculated with the *mll2707/mlr0970* double mutant respect to those inoculated with the *mlr0970* single mutant was significant (*p* < 0.05 according to Student’s *t*-test). This indicates that the mutation of *mll2707* over the *mlr0970* mutant background had an effect on the dry weight of plants. These results, together with the characteristics of the nodules induced by bacteria carrying the mutation in the *mll2707* gene, allow us to conclude that these bacteria induce ineffective nodules.

As described in Section “Materials and Methods,” the occupancy degree reached by the different strains was determined by counting the number of viable bacteria located within the nodule at 45 days post inoculation. As shown in Table 2, the *tatC* mutant strain presented a very low occupancy level. The *mll2707* and *mll2707*/*mlr0970* mutants showed a slight lower occupancy level than the wild-type strain but much higher than the *tatC* mutant strain.

**Table 2:** Nodule occupancy analysis of Rieske mutants.

*Strain*	*N° cells/nodule*	*SD*	
*tatC*	3.8 × 10^2^	8.3 × 10^1^	*a*
*2707/0970*	7.6 × 10^5^	1.5 × 10^5^	*b*
*2707*	1.0 × 10^6^	2.6 × 10^5^	*b*
*0970*	2.2 × 10^6^	7.5 × 10^5^	*c*
Wild-type	1.1 × 10^7^	9.1 × 10^5^	*d*


*For each bacterial strain, six nodules were surface sterilized and plated individually on AB medium. Data was analyzed by Kruskal–Wallis and Mann–Whitney pairwise comparisons. Different letters indicate significant differences: *a*, differs from *b*, *c*, and *d*, at *p* < 0.01; *b* differs from *c* at *p* < 0.05; *c* differs from *d* at *p* < 0.05; *b* differs from *d* at *p* < 0.01. SD, standard deviations from replicates.*

## Discussion

*Mesorhizobium loti* MAFF303099 genome encodes for two Rieske Fe/S proteins. The *mll2707* and *mlr0970* genes, coding for the respective Rieske proteins, presented a different transcriptional expression pattern. The *mll2707* gene is expressed constitutively meanwhile the *mlr0970* expression could be detected only under *in vitro* anaerobic or low oxygen conditions and in nodules. Each Rieske Fe/S gene is localized in its respective cytochrome bc_1_-type cluster. Several bacteria present two or more copies of the Rieske Fe/S coding genes, but not all of them present this gene placed within the cluster of genes that code for the Rieske/Cyt bc_1_-type complex (Schneider and Schmidt, 2005; Ten Brink et al., 2013). *M. loti* MAFF303099 is the first rhizobia in which the presence of two Rieske proteins is reported. Rieske Fe/S protein has been described to be translocated by the Tat system (Bachmann et al., 2006; De Buck et al., 2007). Both *M. loti* MAFF303099 Rieske Fe/S proteins present a Tat secretion signal, suggesting that both proteins are substrates of the Tat system. Moreover, synthesis or stability of Mll2707 protein was negatively affected in the *tatC* mutant strain. This could not be attributed to a pleiotropic effect of the *tatC* mutation on proteins stability since no effect on Mlr0970 protein expression was observed. It was previously described that mutant strains affected in *tat* genes show a reduction in the protein level for some Tat substrates, not only in the periplasmic space but also in the cytoplasm (Angelini et al., 2001; DeLisa et al., 2003). TatC has been proposed to function both as the principal component of the translocation channel and as a receptor for preproteins (DeLisa et al., 2002; Frobel et al., 2012a). The localization analysis for Mlr0970 suggests that the presence of TatC favored its membrane localization. Since results suggest that *tatC* mutation altered membrane integrity (increased protein secretion to the culture supernatant and SDS sensitivity), to confirm that the translocation of *M. loti* MAFF303099 Rieske Fe/S proteins occurs through this system, experiments with proteins affected by point mutation in their N-terminal twin arginine signal motif should be carried out. Expression of *mll2707* and *mlr0970* genes was negatively regulated in the *tatC* mutant strain. It was previously described in *Pseudomonas aeruginosa* that mutation of *tatC* gene affects the transcriptional expression of some genes, although none of the corresponding proteins were substrate of the Tat system and no molecular mechanism for this regulation was proposed (Ball et al., 2016). In *Yersinia pseudotuberculosis* it was described that mutation in *tatC* gene altered expression of genes related to virulence, stress response and metabolism (Avican et al., 2016).

In some bacteria, such as *Rhodospirillum rubrum* and *Rubrivivax gelatinosus*, that have more than one copy of the gene coding for Rieske Fe/S protein, with only one of them being part of the Rieske/cytochrome bc_1_ cluster, the expressed proteins present functional redundancy (Ouchane et al., 2005; Schneider and Schmidt, 2005). In other bacterial species, such as *Acidithiobacillus ferrooxidans*, which code for two different Rieske/cytochrome bc_1_ complexes, it was determined that the two complexes operate in opposite direction (Schneider and Schmidt, 2005). In our case there are two Rieske/Cyt bc_1_-type clusters but in vegetative *Mesorhizobium* cells the cytochrome bc_1_ functionality depends only on Mll2707 protein activity. Also, according to the nodulation results, only the Mll2707 protein is relevant for the induction of effective nodules. Nodules induced by the mutant strains affected in the *mll2707* gene, although occupied by bacteria, are small and colorless, and appear in a higher number than those induced by the wild-type strain. Data also indicate a reduction in the dry weight of the plants. All these phenotypes have been related to an ineffective nodulation (Leigh et al., 1985; Meloni et al., 2003; D’Antuono et al., 2005). This is in accordance with the previous description of the Rieske Fe/S protein requirement for nitrogen fixation in *B. japonicum* (Thöny-Meyer et al., 1989) and *S. meliloti* (Pickering et al., 2012). Although the expression of the *mlr0970* gene could be detected in nodules, the Mlr0970 function does not appear to be necessary for the nitrogen fixation process at least up to 45 dpi. The only observable defect in the nodules induced by the mutant strain *mlr0970* was a slight but significant reduction in the nodule occupancy level with respect to those induced by the wild-type strain. This difference was less than that presented by the mutants affected in the *mll2707* gene and did not have an apparent consequence in the appearance of the nodules, the number or kinetics of nodulation. In the future, an additional analysis could be carried out to clarify the importance of maintaining this second copy for some other stage of the nodulation process, such as par example the stage of senescence.

Results obtained for cytochrome oxidase assay indicate that TatC is required for cytochrome c-dependent respiration, which is consistent with the lower transcriptional expression of the *mll2707* gene and the absence of Mll2707 reporter protein in the *tatC* mutant strain. Nodulation phenotype of *tatC* mutant strain could be explained largely by defects in the functionality of Mll2707; however, TatC mutation affects the nodulation process in an earlier stage than mutation in the *mll2707* mutation since nodule occupancy by viable bacteria was drastically more affected in the case of the *tatC* mutant strain. This also was previously described for *R. leguminosarum* bv. viciae UPM791 when comparing the nodulation phenotype of *tat* and *cytochrome c_1_* mutant strains (Meloni et al., 2003). It was described in several systems that TatC protein is essential for the function of Tat system (Lavander et al., 2006; González et al., 2007). Since several proteins are translocated by the Tat system, the miss-localization of some of them could be related to this affected symbiotic phenotype. For instance, it was described that the cellulase CelC2 from *R. leguminosarum* is required for nodule invasion (Robledo et al., 2008). Among the high scoring *M. loti* MAFF303099 Tat substrates predicted by the Pred-TAT method, a protein with predicted cellulase activity was found. It could be involved in the first step of root hair invasion; however, it belongs to a different glycosyl hydrolase family than the CelC2 protein. Further confirmation of cellulase translocation by *M. loti* MAFF303099 Tat system and determination of its role in the nodulation process must be carried out. *M. loti* MAFF303099 *tatC* mutant strain shows an increase in supernatant protein secretion. This was previously described for a *tat* mutant of *R. leguminosarum* 3481 strain (Krehenbrink and Downie, 2008). The higher protein secretion and the greater sensitivity to SDS could account for membrane integrity alteration and this could also affect the hair root invasion. It was proposed that in the *tat* mutant of *E. coli* and *R. leguminosarum* a miss-localization of cell wall amidases, normally translocated by the Tat system, causes the outer membrane instability (Stanley et al., 2001; Krehenbrink and Downie, 2008). Among the putative *M. loti* MAFF303099 Tat substrates there are also an amidase and two transpeptidases that could be involved in cell wall integrity. In other rhizobia it was described that Tat mutation has pleiotropic effects on cell division, protein secretion, and sensitivity to antibacterial compounds affecting in consequence the cell viability (Pickering and Oresnik, 2010), or the nodule invasion (Krehenbrink and Downie, 2008).

## Conclusion

In conclusion, this is the first report for a rhizobial strain of the existence of two Rieske Fe/S proteins, coded by genes localized in two different Rieske/Cyt bc_1_-type clusters, which expression depends on the expression of a Tat system component and are differentially regulated by oxygen conditions during growth. Finally, in spite of both genes were transcriptionally expressed in nodules, only the mutation of *mll2707* gene has affected the induction of effective nodules.

## Author Contributions

LAB carried out all the laboratory experiments. AZ assisted in the development of some experiments and in the bioinformatic analysis. LAB, GB, and VCL participated in the discussion and interpretation of results. VCL conceived the idea, designed, and directed the experimental work. VCL wrote the manuscript with collaboration of LAB. All the authors participated in the manuscript revision.

## Conflict of Interest Statement

The authors declare that the research was conducted in the absence of any commercial or financial relationships that could be construed as a potential conflict of interest.

## Supplementary Material

Figure S1 Twin arginine translocation system of *M. loti* MAFF303099.

Figure S2 Analysis of *M. loti* MAFF303099 Rieske sequences. **(A)** Rieske-cytochrome bc_1_ complexes present in *M. loti* MAFF303099 genome. **(B)** Sequence alignment of Mll2707 and Mlr0970 aminoacidic sequences performed by Clustal. The TAT motif containing the Twin Arginine residues are highlighted in gray. The conserved residues coordinating the [2Fe-2S] cluster are underlined.

Figure S3 Nucleotide sequence of *mlr0970* promoter region. The start codon is shown with an angled arrow and putative motif recognized by Fnr-type transcriptional regulator is underlined.

Figure S4 Differential localization of Mlr0970-3XFLAG in wild-type and *tatC* mutant strains. Western blot probed with anti-FLAG antibodies of cytoplasmic and membrane fractions of *M. loti* MAFF303099 *tatC* mutant and wild-type strains containing the *mlr0970* gene fused to a 3XFLAG in an expression vector (pBBRMCS-4). M, protein marker. Positions of size markers loaded onto the gel are labeled (in kDa).

Table S1 Bacterial strains, plasmids, and primers used in this study.

Table S2 Putative TAT-substrates predicted by PRED-TAT software: http://www.compgen.org/tools/PRED-TAT/supplement/genomes Organism: *Mesorhizobium loti* MAFF303099. *M. loti* ID correspond to Rhizobase (*genome.microbedb.jp/rhizobase)*.

Table S3 Relative expression of cytochrome c oxidase FixN genes. The levels of induction of each FixN gene was calculated by Real Time RT-PCR assays using the 2^-(ΔΔ*CT*)^ method. *mll6630* and *mlr6411* genes are located outside and inside the *M. loti* MAFF303099 symbiotic island, respectively.

## References

[B1] Alexandre A. Laranjo M. Oliveira S. (2014). Global transcriptional response to heat shock of the legume symbiont *Mesorhizobium loti* MAFF303099 comprises extensive gene downregulation. *DNA Res.* 21 195–206. 10.1016/j.resmic.2016.07.006 24277738 PMC3989490

[B2] Ames G. Prody C. Kustu S. (1984). Simple, rapid, and quantitative release of periplasmic proteins by chloroform. *J. Bacteriol.* 160 1181–1183. 6501229 10.1128/jb.160.3.1181-1183.1984 PMC215841

[B3] Angelini S. Moreno R. Gouffi K. Santini C. L. Yamagishi A. Berenguer J. (2001). Export of *Thermus thermophilus* alkaline phosphatase via the twin-arginine translocation pathway in *Escherichia coli*. *FEBS Lett.* 506 103–107. 10.1016/S0014-5793(01)02890-3 11591380

[B4] Avican U. Beckstette M. Heroven A. K. Lavander M. Dersch P. Forsberg A. (2016). Transcriptomic and phenotypic analysis reveals new functions for the Tat pathway in *Yersinia pseudotuberculosis*. *J. Bacteriol.* 198 2876–2886. 10.1128/JB.00352-16 27501981 PMC5038016

[B5] Bachmann J. Bauer B. Zwicker K. Ludwig B. Anderka O. (2006). The Rieske protein for *Paracoccus denitrificans* inserted into the cytoplasmic membrane by the twin-arginine translocase. *FEBS J.* 273 4817–4830. 10.1111/j.1742-4658.2006.05480.x 16987314

[B6] Bagos P. G. Nikolaou E. P. Liakopoulos T. D. Tsirigos K. D. (2010). Combined prediction of Tat and Sec signal peptides with hidden Markov models. *Bioinformatics* 26 281–2817. 10.1093/bioinformatics/btq530 20847219

[B7] Ball G. Antelmann H. Imbert P. R. C. Gimenez M. R. Voulhoux R. Ize B. (2016). Translocation system to the exoproteome of *Pseudomonas aeruginosa*. *Sci. Rep.* 6:27675. 10.1038/srep27675 27279369 PMC4899797

[B8] Baymann F. Schoepp-Cothenet B. Lebrun E. van Lis R. Nitschke W. (2012). Phylogeny of Rieske/cytb complexes with a special focus on the haloarchaeal enzymes. *Genome Biol. Evol.* 4 832–841. 10.1093/gbe/evs056 22798450 PMC3509893

[B9] Broughton W. J. Dilworth M. J. (1971). Control of leghemoglobin synthesis in snake beans. *Biochem. J.* 125 1075–1080. 10.1042/bj1251075 5144223 PMC1178271

[B10] Bueno E. Mesa S. Bedmar E. J. Richardson D. J. Delgado M. J. (2012). Bacterial adaptation of respiration from oxic to micooxic and anoxic conditions: redox control. *Antioxid. Redox Signal.* 16 819–852. 10.1089/ars.2011.4051 22098259 PMC3283443

[B11] D’Antuono A. L. Casabuono A. Couto A. Ugalde R. A. Lepek V. C. (2005). Nodule development induced by *Mesorhizobium loti* mutant strains affected in polysaccharide synthesis. *Mol. Plant Microbe Interact.* 18 446–457. 10.1094/MPMI-18-0446 15915643

[B12] De Buck E. Vranckx L. Meyen E. Maes L. Vandersmissen L. Anné J. (2007). The twin-arginine translocation pathway is necessary for correct membrane insertion of the Rieske Fe/S protein in *Legionella pneumophila*. *FEBS Lett.* 581 259–264. 10.1016/j.febslet.2006.12.022 17188684

[B13] de Lorenzo V. Timmis K. N. (1994). Analysis and construction of stable phenotypes in Gram-negative bacteria with Tn5- and Tn10-derived mini-transposons. *Methods Enzymol.* 235 386–405. 10.1016/0076-6879(94)35157-0 8057911

[B14] DeLisa M. P. Tullman D. Georgiou G. (2003). Folding quality control in the export of proteins by the bacterial twin-arginine translocation pathway. *Proc. Natl. Acad. Sci. U.S.A.* 100 6115–6120. 10.1073/pnas.0937838100 12721369 PMC156335

[B15] DeLisa M. P. Samuelson P. Palmer T. Georgiou G. (2002). Genetic analysis of the twin-arginine translocator secretion pathway in bacteria. *J. Biol. Chem.* 277 29825–29831. 10.1074/jbc.M201956200 12021272

[B16] Ditta G. Stanfield S. Corbin D. Helinski D. (1980). Broad host range DNA cloning system for gram-negative bacteria: construction of a gene bank of *Rhizobium meliloti*. *Proc. Natl. Acad. Sci. U.S.A.* 77 7347–7351. 10.1073/pnas.77.12.7347 7012838 PMC350500

[B17] Douglas C. J. Staneloni R. J. Rubin R. A. Nester E. W. (1985). Identification and genetic analysis of an *Agrobacterium tumefaciens* chromosomal virulence region. *J. Bacteriol.* 161 850–860. 2982791 10.1128/jb.161.3.850-860.1985 PMC214975

[B18] Duval S. Santini J. M. Nitschke W. Hille R. Schoepp-Cothenet B. (2010). The small subunit AroB of arsenite oxidase, lessons on the [2Fe-2S] Rieske protein superfamily. *J. Biol. Chem.* 285 20442–20451. 10.1074/jbc.M110.113811 20421651 PMC2898366

[B19] Frobel J. Rose P. Lausberg F. Blummel A. S. Freudl R. Müller M. (2012a). Transmembrane insertion of twin-arginine signal peptides is driven by TatC and regulated by TatB. *Nat. Commun.* 3:1311. 10.1038/ncomms2308 23250441 PMC3538955

[B20] Frobel J. Rose P. Müller M. (2012b). Twin-arginine-dependent translocation of folded proteins. *Phil. Trans. R. Soc. B Biol. Sci.* 367 1029–1046. 10.1098/rstb.2011.0202 22411976 PMC3297433

[B21] González E. T. Brown D. G. Swanson J. K. Allen C. (2007). Using the *Ralstonia solanacearum* Tat secretome to identify bacterial wilt virulence factors. *Appl. Environ. Microbiol.* 73 3779–3786. 10.1128/AEM.02999-06 17468289 PMC1932711

[B22] Kaneko T. Nakamura Y. Sato S. Asamizu E. Kato T. Sasamoto S. (2000). Complete genome structure of the nitrogen-fixing bacterium *Mesorhizobium loti*. *DNA Res.* 7 331–338. 10.1093/dnares/7.6.331 11214968

[B23] Kovach M. E. Elzer P. H. Hill D. S. Robertson G. T. Farris M. A. Martin Roop R. II. (1995). Four new derivatives of the broad-host-range cloning vector pBBR1MCS, carrying different antibiotic-resistance cassettes. *Gene* 166 175–176. 10.1016/0378-1119(95)00584-1 8529885

[B24] Krehenbrink M. Downie J. A. (2008). Identification of protein secretion systems and novel secreted proteins in *Rhizobium leguminosarum* bv. *viciae. BMC Genomics* 9:55. 10.1186/1471-2164-9-55 18230162 PMC2275737

[B25] Lavander M. Ericsson S. K. Broms J. E. Forsberg A. (2006). The twin arginine translocation system is essential for virulence of *Yersinia pseudotuberculosis*. *Infect. Immun.* 74 1768–1776. 10.1128/IAI.74.3.1768-1776.2006 16495550 PMC1418654

[B26] Leigh J. A. Signer E. R. Walker G. C. (1985). Exopolysaccharide-deficient mutants of *Rhizobium meliloti* that form ineffective nodules. *Proc. Natl. Acad. Sci. U.S.A.* 82 6231–6235 10.1073/pnas.82.18.6231 3862129 PMC391026

[B27] Livak K. J. Schmittgen T. D. (2001). Analysis of relative gene expression data using real-time quantitative PCR and the 2(-Delta Delta C(T)) Method. *Methods* 25 402–408. 10.1006/meth.2001.1262 11846609

[B28] Martínez-Hidalgo P. Ramírez-Bahena M. H. Flores-Félix J. D. Igual J. M. Sanjuán J. León-Barrios M. (2016). Reclassification of strains MAFF 303099^T^ and R7A into *Mesorhizobium japonicum* sp. nov. *Int. J. Syst. Evol. Microbiol.* 66 1–6. 10.1099/ijsem.0.001448 27565417

[B29] Meloni S. Rey L. Sidler S. Imperial J. Ruiz-Argüeso T. Palacios J. M. (2003). The twin-arginine translocation (Tat) system is essential for *Rhizobium-legume* symbiosis. *Mol. Microbiol.* 48 1195–1207. 10.1046/j.1365-2958.2003.03510.x 12787349

[B30] Mercante V. Duarte M. C. Sánchez C. M. Zalguizuri A. Caetano-Anollés G. Lepek V. C. (2015). The absence of protein Y4yS affects negatively the abundance of T3SS *Mesorhizobium loti* secretin, RhcC2, in bacterial membranes. *Front. Plant Sci.* 6:12. 10.3389/fpls.2015.00012 25688250 PMC4311626

[B31] Okazaki S. Okabe S. Higashi M. Shimoda Y. Sato S. Tabata S. (2010). Identification and functional analysis of type III effector proteins in *Mesorhizobium loti*. *Mol. Plant Microbe Interact.* 23 223–234. 10.1094/MPMI-23-2-0223 20064065

[B32] Ouchane S. Nitschke W. Blanco P. Vermegilo A. Astier C. (2005). Multiple Rieske genes in prokaryotes: exchangeable Rieske subunits in the cytochrome bc1-complex of *Rubrivivax gelatinosus*. *Mol. Microbiol.* 57 261–275. 10.1111/j.1365-2958.2005.04685.x 15948965

[B33] Pfaffl M. W. (2001). A new mathematical model for relative quantification in real-time RT–PCR. *Nucleic Acid Res.* 129 2003–2007. 10.1093/nar/29.9.e45 PMC55695 11328886

[B34] Pickering B. S. Oresnik I. J. (2010). The twin arginine transport system appears to be essential for viability in *Sinorhizobium meliloti.* *J. Bacteriol.* 192 5173–5180. 10.1128/JB.00206-10 20675496 PMC2944528

[B35] Pickering B. S. Yudistira H. Oresnik I. J. (2012). Characterization of the twin-arginine transport secretome in *Sinorhizobium meliloti* and evidence for host-dependent phenotypes. *Appl. Environ. Microbiol.* 78 7141–7144. 10.1128/AEM.01458-12 22843517 PMC3457499

[B36] Robledo M. Jiménez-Zurdo J. I. Velázquez E. Trujillo M. E. Zurdo-Piñeiro J. L. Ramírez-Bahena M. H. (2008). Rhizobium cellulase CelC2 is essential for primary symbiotic infection of legume host roots. *Proc. Natl. Acad. Sci. U.S.A.* 105 7064–7069. 10.1073/pnas.0802547105 18458328 PMC2383954

[B37] Sánchez C. Iannino F. Deakin W. J. Ugalde R. A. Lepek V. C. (2009). Characterization of the *Mesorhizobium loti* MAFF303099 type three protein secretion system. *Mol. Plant Microbe Interact.* 22 519–528. 10.1094/MPMI-22-5-0519 19348570

[B38] Sánchez C. Mercante V. Babuin M. F. Lepek V. C. (2012). Dual effect of *Mesorhizobium loti* T3SS functionality on the symbiotic process. *FEMS Microbiol. Lett.* 330 148–156. 10.1111/j.1574-6968.2012.02545.x 22428564

[B39] Sargent F. Bogsch E. G. Stanley N. R. Wexler M. Robinson C. Berks B. C. (1998). Overlapping functions of components of a bacterial Sec-independent protein export pathway. *EMBO J.* 17 3640–3650. 10.1093/emboj/17.13.3640 9649434 PMC1170700

[B40] Schäfer A. Tauch A. Jäger W. Kaninowski J. Thierbach G. Pühler A. (1994). Small mobilizable multi-purpose cloning vectors derived from the *Escherichia coli* plasmids pK18 and pK19: selection of defined deletions in the chromosome of *Corynebacterium glutamicum*. *Gene* 145 69–73. 10.1016/0378-1119(94)90324-7 8045426

[B41] Schmeisser C. Liesegang H. Krysciak D. Bakkou N. Le Quéré A. Wollherr A. (2009). Rhizobium sp. strain NGR234 possesses a remarkable number of secretion systems. *Appl. Environ. Microbiol.* 75 4035–4045. 10.1128/AEM.00515-09 19376903 PMC2698369

[B42] Schneider D. Schmidt C. L. (2005). Multiple Rieske proteins in prokaryotes: where and why? *Biochem. Biophys. Acta* 1710 1–12. 10.1016/j.bbabio.2005.09.003 16271700

[B43] Simone D. Day D. C. Leach T. Turner R. J. (2013). Diversity and evolution of bacterial twin arginine translocase protein, TatC, reveals a protein secretion system that is evolving to fit its environmental niche. *PLoS One* 8:e78742. 10.1371/journal.pone.0078742 24236045 PMC3827258

[B44] Spano S. Ugalde J. E. Galán J. E. (2008). Delivery of a *Salmonella typhi* exotoxin from a host intracellular compartment. *Cell Host Microbe* 3 30–38. 10.1016/j.chom.2007.11.001 18191792

[B45] Stanley N. R. Findlay K. Berks B. C. Palmer T. (2001). *Escherichia coli* strains blocked in Tat-dependent protein export exhibit pleiotropic defects in the cell envelope. *J. Bacteriol.* 183 139–144. 10.1128/JB.183.1.139-144.2001 11114910 PMC94859

[B46] Sturz A. V. Christie B. R. Matheson B. G. Nowak J. (1997). Biodiversity of endophytic bacteria which colonize red clover nodules, roots, stems and foliage and their influence on host growth. *Biol. Fertil. Soils* 25 13–19. 10.1007/s003740050273

[B47] Ten Brink F. Schoepp-Cothenet B. van Lis R. Nitschke W. Baymann F. (2013). Multiple rieske/Cytb complexes in a single organism. *Biochim. Biophys. Acta* 1827 1392–1406. 10.1016/j.bbabio.2013.03.003 23507620

[B48] Thöny-Meyer L. Stax D. Hennecke H. (1989). An unusual gene cluster for the cytochrome bc1 complex in *Bradyrhizobium japonicum* and its requirement for effective root nodule symbiosis. *Cell* 57 683–697. 10.1016/0092-8674(89)90137-2 2541921

[B49] Uchiumi T. Ohwada T. Itakura M. Mitsui H. Nukui N. Dawadi P. (2004). Expression islands clustered on the simbiosis island of the *Mesorhizobium loti* genome. *J. Bacteriol.* 186 2439–2448. 10.1128/JB.186.8.2439-2448.2004 15060047 PMC412173

[B50] Ugalde J. E. Czibener C. Feldman M. F. Ugalde R. A. (2000). Identification and characterization of the *Brucella abortus* phosphoglucomutase gene: role of lipopolysaccharide in virulence and intracellular multiplication. *Infect. Immun.* 68 5716–5723. 10.1128/IAI.68.10.5716-5723.2000 10992476 PMC101528

[B51] Vincent J. M. (1970). *A Manual for the Practical Study of Root Nodule Bacteria.* Oxford: Blackwell Scientific Publications.

[B52] Woodcock D. H. Crowther P. J. Doherty J. Jefferson S. DeCruz E. Noyer-Weidner M. (1989). Quantitative evaluation of *Escherichia coli* host strains for tolerance to cytosine methylation in plasmid and phage recombinants. *Nucleic Acids Res.* 17 3469–3478. 10.1093/nar/17.9.3469 2657660 PMC317789

[B53] Wu G. Delgado M. J. Vargas C. Davies A. E. Poole R. K. Downie J. A. (1996). The cytochrome bc1 complex but not CycM is necessary for symbiotic nitrogen fixation by *Rhizobium leguminosarum*. *Microbiology* 142 2281–3388. 10.1099/13500872-142-12-3381 9004501

