# Supplementary figures and images for: Two Rieske Fe/S Proteins and TAT System in Mesorhizobium loti MAFF303099: Differential Regulation and Roles on Nodulation
Source: Front Plant Sci. 2018 Nov 20;9:1686. doi: 10.3389/fpls.2018.01686 (PMC6256036; doi:10.3389/fpls.2018.01686)

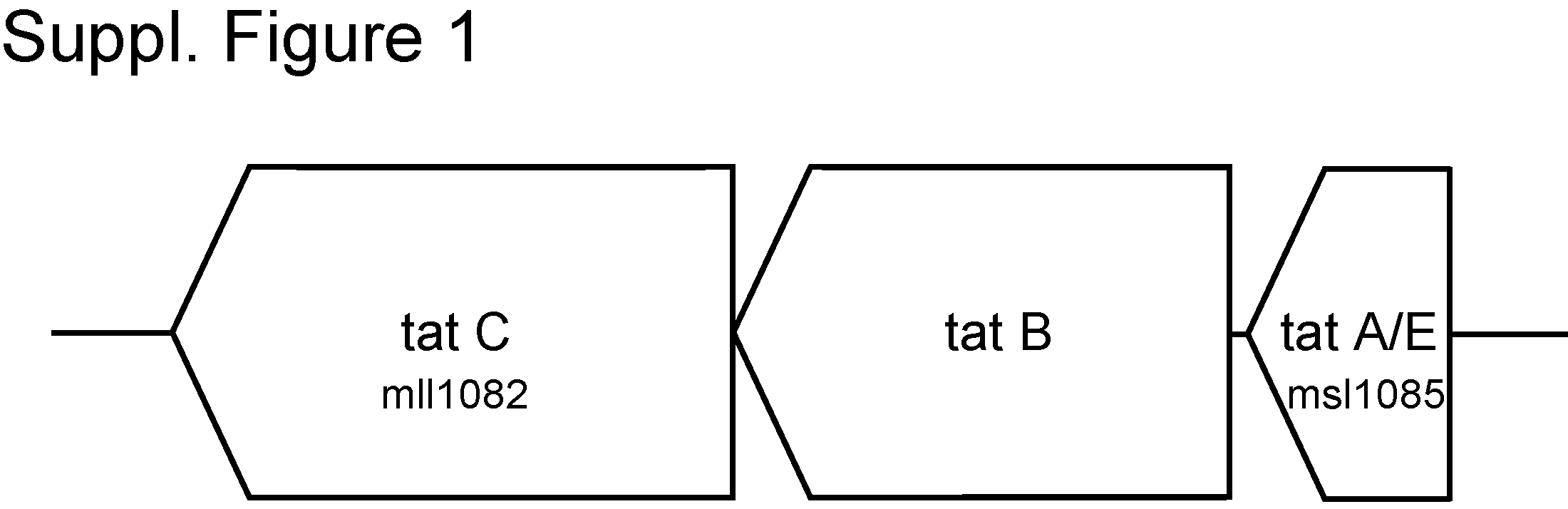

Supplement: Figure S1 — Twin arginine translocation system of M. loti MAFF303099. [file Image_1.TIF]

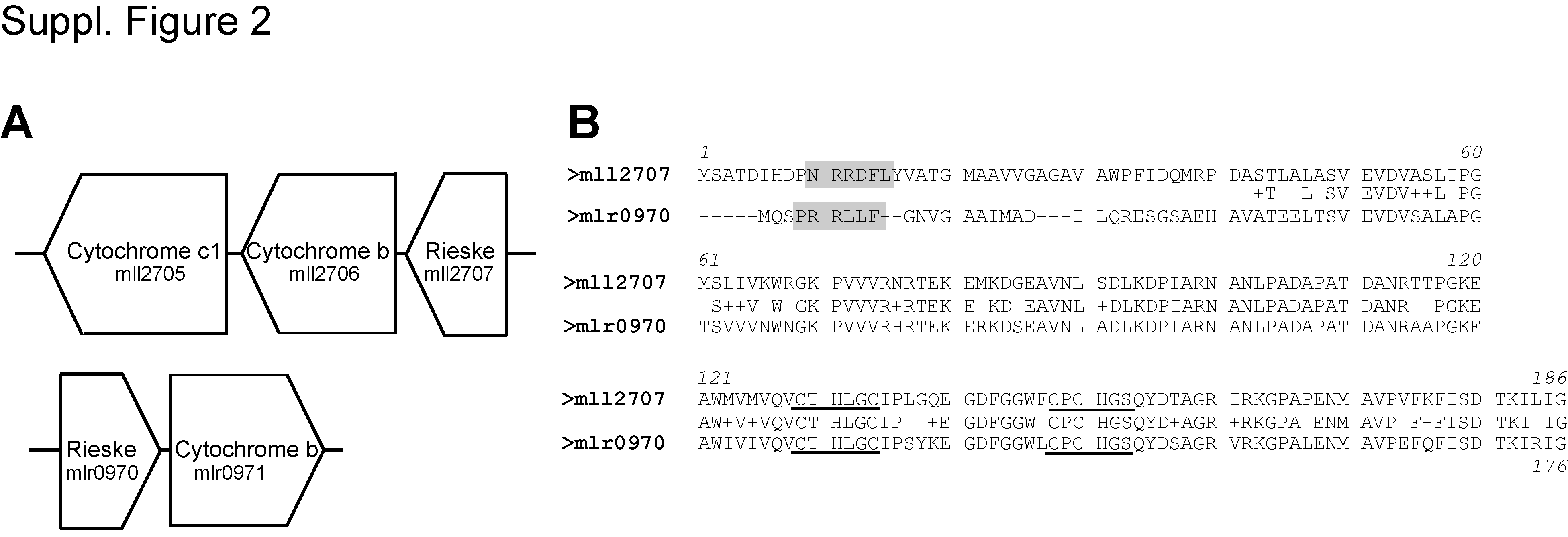

Supplement: Figure S2 — Analysis of M. loti MAFF303099 Rieske sequences. (A) Rieske-cytochrome bc1 complexes present in M. loti MAFF303099 genome. (B) Sequence alignment of Mll2707 and Mlr0970 aminoacidic sequences performed by Clustal. The TAT motif containing the Twin Arginine residues are highlighted in gray. The conserved residues coordinating the [2Fe-2S] cluster are underlined. [file Image_2.TIF]

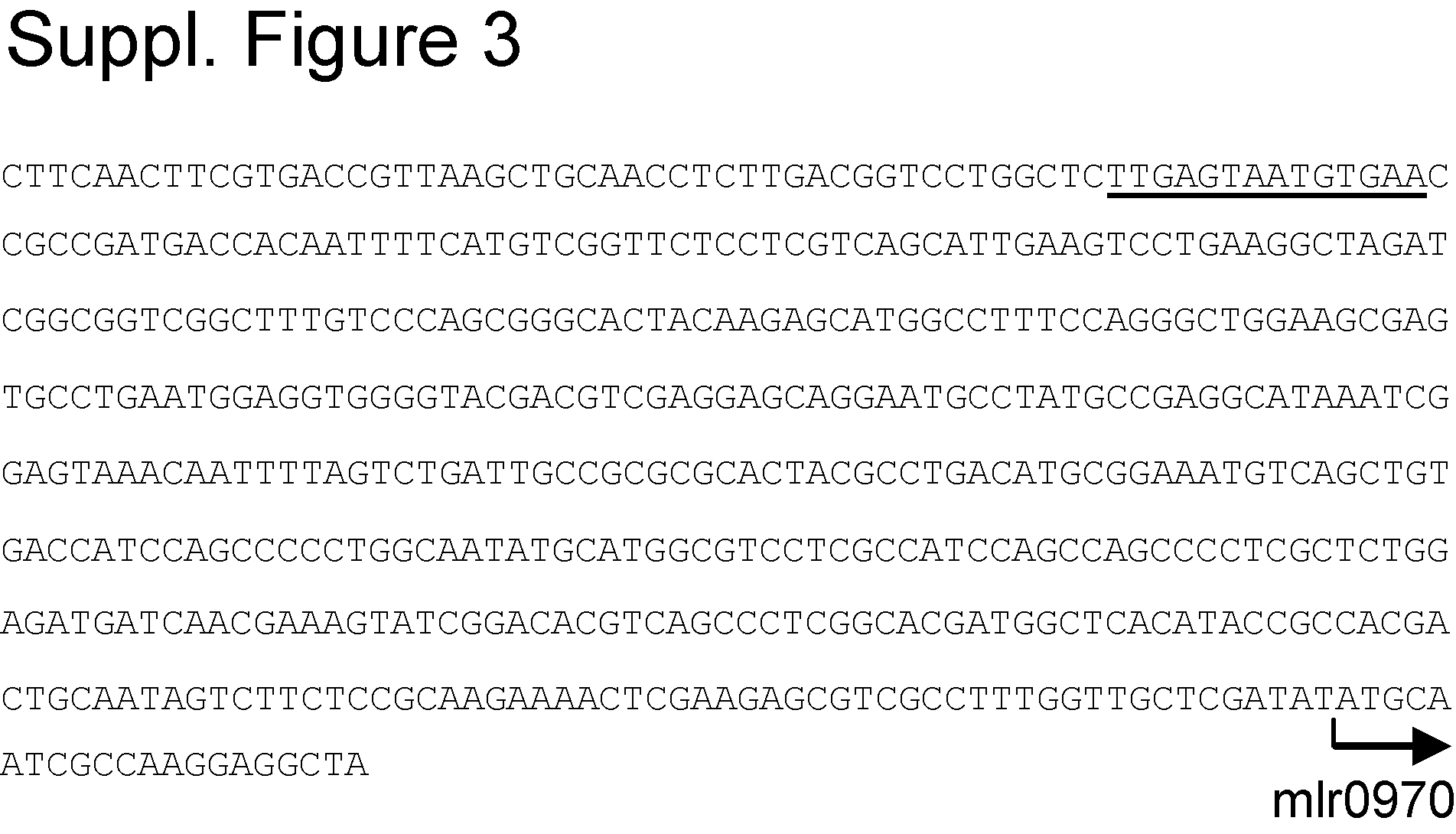

Supplement: Figure S3 — Nucleotide sequence of mlr0970 promoter region. The start codon is shown with an angled arrow and putative motif recognized by Fnr-type transcriptional regulator is underlined. [file Image_3.TIF]

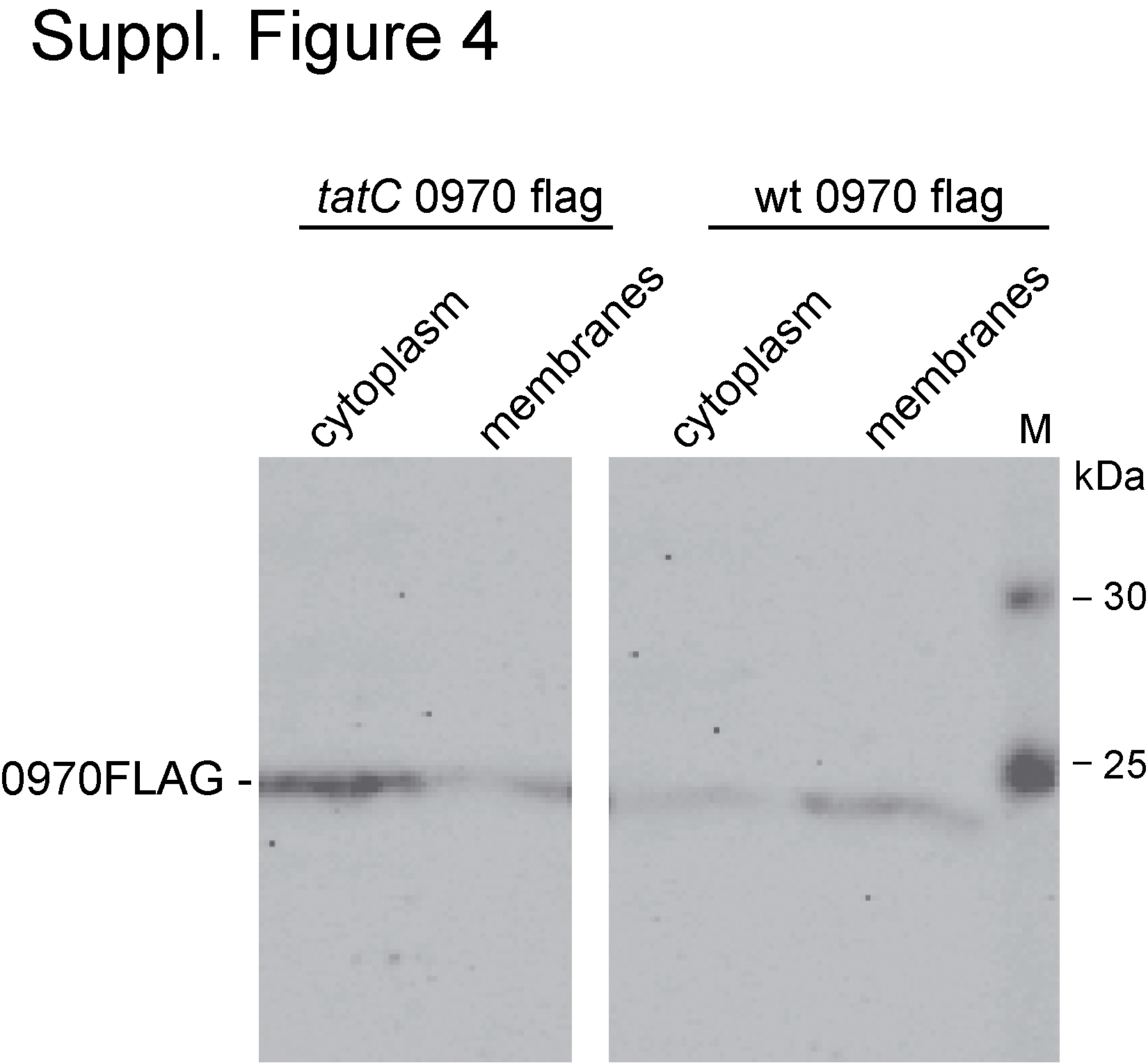

Supplement: Figure S4 — Differential localization of Mlr0970-3XFLAG in wild-type and tatC mutant strains. Western blot probed with anti-FLAG antibodies of cytoplasmic and membrane fractions of M. loti MAFF303099 tatC mutant and wild-type strains containing the mlr0970 gene fused to a 3XFLAG in an expression vector (pBBRMCS-4). M, protein marker. Positions of size markers loaded onto the gel are labeled (in kDa). [file Image_4.TIF]
