# Supplementary material for: Two Rieske Fe/S Proteins and TAT System in Mesorhizobium loti MAFF303099: Differential Regulation and Roles on Nodulation
Source: Front Plant Sci. 2018 Nov 20;9:1686. doi: 10.3389/fpls.2018.01686 (PMC6256036; doi:10.3389/fpls.2018.01686)
Supplement: Table S1 — Bacterial strains, plasmids, and primers used in this study. [file Table_1.DOC]

**Supplemental Table 1.** **Bacterial strains, plasmids, and primers used in this study**

|  | **Genotype or characteristics** | **Reference or source** |
| --- | --- | --- |
| **Strains** | | |
| ***E.coli* K-12** |  |  |
| DH5α-F’IQ | F´ Φ80d lacZΔM15 Δ(lacZYA-argF) U169 deoR recA1 endA1 hsdR17 (rK – mK^+^) phoA supE44 λ^-^ thi-1 gyrA96 relA1/F´proAB^+^ lacI^q^Z ΔM15 zzf::Tn5[Km^r^] | Woodcock et al., 1989 |
| S17-1 λpir | λ lysogenic S17-1 derivative producing π protein for replication of  plasmids carrying *oriR6K* | de Lorenzo V. &  Timmis K. N., 1994 |
| ***M. loti*** |  |  |
| MAFF303099 | Wild-type, Nod^+^ | Kaneko et al., 2000a,b |
| *tatC* | *tatC*:: Gm , Gm^r^ | This study |
| *tatC* plus tatC | *tatC*:: Gm , Gm^r^, *tatC* in pBBR1MCS-2 expression plasmid, Km^r^ | This study |
| *mll2707* | *mll2707::*Gm^r^ | This study |
| *mlr0970* | *mlr0970::*Tc^r^ | This study |
| *mll2707*/ *mlr0970* | *mll2707::*Gm^r^ , *mlr0970::*Tc^r^ | This study |
| wt 2707-flag | MAFF303099 with *mll2707* coding sequence translationally fused to 3XFLAG in pBBR1MCS-4 expression plasmid, Amp^r^ | This study |
| wt 0970-flag | MAFF303099 with *mlr0970* coding sequence translationally fused to 3XFLAG in pBBR1MCS-4 expression plasmid, Amp^r^ | This study |
|    * tatC* 2707-flag | *tatC* strain with *mll2707* coding sequence translationally fused to 3XFLAG in pBBR1MCS-4 expression plasmid, Amp^r^ | This study |
| *    tatC* 0970-flag | *tatC* strain with *mlr0970* coding sequence translationally fused to 3XFLAG in pBBR1MCS-4 expression plasmid, Amp^r^ | This study |
|  * tatC* 2707-flag-plus TatC | *tatC* strain with *mll2707* fused to 3XFLAG in pBBR1MCS-4 expression plasmid, Amp^r^ , and with *tatC* in pBBR1MCS-2 expression plasmid, Km^r^ | This study |
| *tatC* 0970-flag-plus TatC | *tatC* strain with *mlr0970* fused to 3XFLAG in pBBR1MCS-4 expression plasmid, Amp^r^ , and with *tatC* in pBBR1MCS-2 expression plasmid, Km^r^ | This study |
| *mll2707*plus mll2707 | *mll2707* strain with *mll2707* fused to 3XFLAG in pBBR1MCS-4 expression plasmid, Amp^r^ | This study |
| **Plasmids** | | |
| pGEM-TEasy | pGEM®-5Zf(+) derivative, F1 replication origin. Amp^r^ | Promega |
| pSPG1 | Gentamicin cassette, Gm^r^ | Ugalde et al., 2000 |
| pRK2013 | RK2 derivative helper plasmid; Km^r^ | Ditta et al., 1980 |
| pK18*mob* | Mobilizable derivative of pK18, Km^r^ | Schafer et al., 1994 |
| pK18*mob*Tc | pK18*mob* vector containing a Tetracycline cassette, Km^r^ Tc^r^ | Sánchez et al., 2009 |
| pBBR1MCS-4 | Broad-host-range cloning vector. Amp^r^ | Kovach M., 1995 |
| pBBR1MCS-2 | Broad-host-range cloning vector. Km^r^ | Kovach M., 1995 |
| pBAD24 3xFLAG | Region encoding 3x FLAG epitope in pBAD24, Amp^r^ | Spano et al., 2008 |
| **Primers** | | |
| **Primers for mutants construction** |  |  |
| TatC-F | 5´ ggacaaggaaaaggacgag 3´ | This study |
| TatC-Xba-R | 5´tctagacgacgaaagcaatgacga 3´ | This study |
| 2707-F | 5´ttgtgcggcgtatgcggt 3´ | This study |
| 2707-Xba-R  0970-F1 | 5´tctagagccttgtaggagccgtaa 3´  5´ccagatacaggaggcgac 3´ | This study |
| 0970-Bam-R1 | 5´ggatccaggtcagttcctcggtgg 3´ | This study |
| 0970-Bam-F2 | 5´ggatccgacctcaaggacccgatt 3´ | This study |
| 0970-Xba-R2 | 5´tctagagccatcataagcagcacg 3´ | This study |
| **Primers for *tatC* complementation** |  |  |
| cTatC-Hind-F | 5´aagcttccaagaagacggcgggag 3´ | This study |
| cTatC-Bam-R | 5´ggatccccgctgacggcttacgaa 3´ | This study |
| **Primers for translational fusion to 3x FLAG** |  |  |
| Fl2707-BglXho-F | 5´ttagatctctcgagcggaagaggcgaaaggat 3´ | This study |
| Fl2707-Eco-R | 5´aattgaattcaaggatcttggtatcgg 3´ | This study |
| Fl0970-Bam-F | 5´aatggatccgagcgtcgcctttggttg 3´ | This study |
| Fl0970-Nco-R | 5´attccatggtgccgatacggattttggt 3´ | This study |
| **Primers for real time PCR analysis** |  |  |
| q2707-F | 5´acctctccgatctcaagga 3´ | This study |
| q0970-F | 5´acctcaaggacccgattg 3´ | This study |
| qRieskes-R | 5´atgcagcccagatgcgtg 3´ | This study |
| qSigA-F | 5´gctttacgacatcaacaagcg 3´ | This study |
| qSigA-R | 5´gagttcggaaccctgatattcc 3´ | This study |
| qRpoA-F | 5´acgacaacatcgtctacatcg 3´ | This study |
| qRpoA-R | 5´acttccttgatctcgttcagc 3´ | This study |
| q6630-F | 5´ctggtcacctggcacttc 3´ | This study |
| q6630-R | 5´gccctgctcgtcgtattc 3´ | This study |
| q6411-F | 5´tccgcctacatggatggc 3´ | This study |
| q6411-R | 5´gatgttgaggtgagggtatg 3´ | This study |
