# Supplementary material for: Two Rieske Fe/S Proteins and TAT System in Mesorhizobium loti MAFF303099: Differential Regulation and Roles on Nodulation
Source: Front Plant Sci. 2018 Nov 20;9:1686. doi: 10.3389/fpls.2018.01686 (PMC6256036; doi:10.3389/fpls.2018.01686)
Supplement: Table S2 — Putative TAT-substrates predicted by PRED-TAT software: http://www.compgen.org/tools/PRED-TAT/supplement/genomes Organism: Mesorhizobium loti MAFF303099. M. loti ID correspond to Rhizobase (genome.microbedb.jp/rhizobase). [file Table_2.DOC]

**Supplemental Table 2. Putative TAT-substrates predicted by PRED-TAT software**

<http://www.compgen.org/tools/PRED-TAT/supplement/genomes>

Organism: *Mesorhizobium loti* MAFF303099. *M. loti* ID correspond to Rhizobase (genome.microbedb.jp/rhizobase).

GI number *M.loti* ID Tat signal (pos.) Description

13470588 msr0341 1-40 hypothetical protein

13470608 mlr0366 1-33 bifunctional 2',3'-cyclic nucleotide 2'-phosphodiesterase/3'-nucleotidase

13470658 mll0429 1-40 hypothetical protein

13470667 msl0439 1-33 hypothetical protein

13470711 mlr0488 1-47 hypothetical protein

13470841 mll0643 1-33 uridylate kinase

13470843 mll0646 1-50 nuclear transport factor 2 family protein

13470933 mlr0764 1-54 dTDP-glucose 4,6-dehydratase

13471018 mlr0879 1-46 molybdopterin oxidoreductase

13471087 mlr0970 1-31 **ubiquinol-cytochrome C reductase iron-sulfur subunit**

13471105 mll0989 1-38 two component sensor-kinase

13471111 mll0995 1-66 secretion protein

13471126 mll1012 1-29 ribose ABC transporter substrate-binding protein

13471169 mll1067 1-49 hypothetical protein

13471181 mll1082 1-47 sec-independent protein translocase component

13471182 mlr1084 1-44 hypothetical protein

13471231 mll1146 1-62 hypothetical protein

13471471 mll1451 1-37 serine protease

13471503 mll1495 1-46 carboxymethyl 1butenolidase

13471521 mll1519 1-45 L,D-transpeptidase

13471578 mlr1592 1-25 hypothetical protein

13471630 mll1660 1-60 peptide ABC transporter substrate-binding protein

13471703 mll1755 1-29 myristoyl transferase

13471817 mll1915 1-51 hypothetical protein

13471903 mlr2023 1-49 spermidine/putrescine ABC transporter substrate-binding protein

13471915 mlr2037 1-33 hypothetical protein

13471952 mlr2086 1-35 cellulase

13471954 mll2090 1-57 lipopolysaccharide biosynthesis protein

13471994 mll2148 1-45 ribose-binding protein of ribose ABC transporter

13472011 mll2168 1-29 aquaporin family protein

13472065 mll2240 1-60 diguanylate cyclase

13472073 mll2250 1-41 histidine kinase

13472092 mll2276 1-66 oligopeptide binding protein of oligopeptide ABC transporter

13472145 mlr2346 1-31 type VI secretion lipoprotein

13472275 mlr2516 1-43 hypothetical protein

13472335 mll2597 1-29 sulfite oxidase

13472358 mll2626 1-37 hypothetical protein

13472419 mll2707 1-48 **ubiquinol-cytochrome C reductase iron-sulfur subunit**

13472471 mlr2777 1-25 intracellular sulphur oxidation protein

13472515 mlr2839 1-45 hypothetical protein

13472519 mll2846 1-70 hypothetical protein

13472531 mlr2858 1-30 nitrate transporter component, nrtA

13472549 mlr2880 1-30 transcriptional regulator

13472588 mlr2932 1-47 chemotaxis MOTC protein

13472844 mlr3265 1-60 peptide ABC transporter substrate-binding protein

13472857 msl3283 1-51 hypothetical protein

13472861 mlr3288 1-37 epoxide hydrolase

13472894 mlr3334 1-33 ribose ABC transporter substrate-binding protein

13472936 mll3386 1-56 transcriptional regulator

13472942 mlr3395 1-30 ABC transporter oligopeptide-binding protein

13472962 mll3424 1-51 transmembrane efflux protein

13473085 mlr3575 1-48 site-specific tyrosine recombinase XerD

13473095 mll3586 1-22 oxidoreductase

13473139 mll3638 1-52 transcriptional regulatory protein

13473164 mll3670 1-28 permidine/putrescine ABC transporter substrate-binding protein

13473287 mll3836 1-35 aldehyde dehydrogenase

13473363 mll3934 1-41 ABC transporter ATP-binding protein

13473498 mll4115 1-34 secreted alkaline phosphatase

13473548 mlr4190 1-30 spermidine/putrescine ABC transporter substrate-binding protein

13473552 mlr4194 1-37 macrolide glycosyltransferase

13473592 mll4243 1-58 DNA translocase FtsK

13473817 mll4539 1-55 dipeptide ABC transporter substrate-binding protein

13474016 mll4794 1-43 outer membrane lipoprotein

13474036 mll4820 1-34 methionine sulfoxide reductase B

13474052 msl4838 1-48 hypothetical protein

13474091 mll4889 1-48 branched-chain amino acid ABC transporter substrate-binding protein

13474270 mll5121 1-45 ABC transporter binding protein

13474274 mll5127 1-46 ABC transporter binding protein

13474308 mlr5166 1-30 periplasmic mannitol ABC transporter substrate-binding protein

13474389 mlr5264 1-51 endo-1,3-1,4-BETA-glycanase EXOK

13474440 mll5321 1-50 uridylyl-transferase

13474523 mll5423 1-26 amino acid ABC-transporter substrate-binding protein

13474538 mll5441 1-29 peptide ABC transporter substrate-binding protein

13474546 mll5452 1-34 hypothetical protein

13474564 mll5471 1-49 arylsulfatase

13474581 mll5493 1-28 ABC transporter substrate-binding protein

13474608 mlr5527 1-54 thiol oxidoreductase

13474610 mlr5529 1-29 hypothetical protein

13474732 mll5686 1-29 transmembrane efflux protein

13474753 mlr5711 1-27 gamma-glutamyltranspeptidase

13474908 mlr5892 1-29 ABC transporter substrate-binding protein

13475072 mlr6078 1-32 ABC transporter binding protein component

13475076 mlr6082 1-43 ABC transporter binding protein component

13475114 mll6123 1-29 5-methyltetrahydropteroyltriglutamate--homocysteine S-methyltransferase

13475193 mlr6217 1-49 2-methylcitrate dehydratase

13475229 mll6256 1-38 ABC transporter binding protein component

13475344 mll6389 1-35 porin

13475359 mlr6405 1-70 conjugal transfer protein trbI

13475409 mll6469 1-25 HylD family type I secretion periplasmic adaptor subunit

13475430 mlr6496 1-33 metallo-oxidoreductase

13475503 mll6590 1-34 cation-transporting ATPase

13475576 mlr6682 1-50 epoxide hydrolase

13475614 mll6731 1-47 multidrug efflux membrane fusion protein

13475664 mlr6788 1-31 2-methyl-3-hydroxypyridine-5-carboxylic acid oxygenase

13475672 mlr6799 1-27 hypothetical protein

13475828 mlr6997 1-37 ABC transporter substrate-binding protein

13475882 mlr7061 1-31 ABC-transporter substrate-binding protein

13476001 mll7204 1-26 ABC transporter substrate binding protein

13476049 mlr7261 1-49 ABC transporter binding protein

13476115 mll7344 1-56 peptide ABC-transporter substrate-binding protein

13476120 mll7351 1-68 ABC transporter binding protein

13476252 mlr7525 1-43 DSBA oxireductase

13476328 mll7617 1-28 hypothetical protein

13476341 mll7632 1-49 L,D-transpeptidase

13476375 mlr7676 1-48 ABC transporter binding protein

13476476 mlr7806 1-39 rarD protein, chloamphenicol sensitive

13476533 mlr7876 1-71 hypothetical protein

13476536 mll7879 1-42 dTDP-glucose 4-6-dehydratase

13476667 mlr8053 1-27 protein-L-isoaspartate O-methyltransferase

13476739 mlr8147 1-30 metal-binding protein

13476760 mlr8178 1-58 metallophosphoesterase

13476803 mll8234 1-47 serine protease

13476842 mlr8282 1-60 diguanylate cyclase

13476889 mlr8341 1-55 hypothetical protein

13476933 mlr8397 1-31 amidase

13476942 mlr8412 1-40 arabinose efflux permease

13476969 mll8444 1-41 ABC transporter binding protein

13477033 mlr8526 1-40 hypothetical protein

13477072 mll8573 1-31 nitrate ABC transporter

13488182 mlr9013 1-37 spermidine/putrecine ABC transporter

13488349 msr9092 1-40 hypothetical protein

13488093 mll9149 1-28 oligopeptide ABC transporter oligopeptide-binding protein

13488116 mll9181 1-34 periplasmic component ABC transporter

13488240 mlr9210 1-46 dipeptide binding protein

13488274 mlr9267 1-29 extracellular solute-binding protein

13488275 mlr9269 1-29 extracellular solute-binding protein

13488571 mlr9745 1-66 lytic transglycosylase

13488538 mlr9704 1-38 methylase/helicase

13488568 mlr9741 1-66 glycosidase
