# Supplementary material for: Two Rieske Fe/S Proteins and TAT System in Mesorhizobium loti MAFF303099: Differential Regulation and Roles on Nodulation
Source: Front Plant Sci. 2018 Nov 20;9:1686. doi: 10.3389/fpls.2018.01686 (PMC6256036; doi:10.3389/fpls.2018.01686)
Supplement: Table S3 — Relative expression of cytochrome c oxidase FixN genes. The levels of induction of each FixN gene was calculated by Real Time RT-PCR assays using the 2-(ΔΔCT) method. mll6630 and mlr6411 genes are located outside and inside the M. loti MAFF303099 symbiotic island, respectively. [file Table_3.DOC]

| **Conditions** | **Gene** | **Relative expression** |
| --- | --- | --- |
| *Double layer vs aerobic plate* | *mll6630* | 2.90 ± 1.16 |
|  | *mlr6411* | 10.08 ± 1.06 |
|  |  |  |
| *Anaerobic vs aerobic plate* | *mll6630* | 26.56 ± 1.37 |
|  | *mlr6411* | 145.80 ± 2.39 |

**Supplemental Table 3:** **Relative expression of Cytochrome c oxidase FixN genes.** The levels of induction of each FixN gene was calculated by Real Time RT-PCR assays using the 2 ^-(ΔΔCT)^ method. *mll6630* and *mlr6411* genes are located outside and inside the *M. loti* MAFF303099 symbiotic island, respectively.
